# Numerical Models Can Assist Choice of an Aortic Phantom for In Vitro Testing

**DOI:** 10.3390/bioengineering8080101

**Published:** 2021-07-21

**Authors:** Giulia Comunale, Luigi Di Micco, Daniela Paola Boso, Francesca Maria Susin, Paolo Peruzzo

**Affiliations:** 1 Cardiovascular Fluid Dynamics Laboratory HER, Department of Civil, Environmental and Architectural Engineering, University of Padova, 35131 Padova, Italy; giulia.comunale@dicea.unipd.it (G.C.); luigi.dimicco@dicea.unipd.it (L.D.M.); francescamaria.susin@dicea.unipd.it (F.M.S.); 2 Department of Civil, Environmental and Architectural Engineering, University of Padova, 35131 Padova, Italy; daniela.boso@dicea.unipd.it

**Keywords:** FSI simulations, patient-specific aorta, aorta’s hemodynamics

## Abstract

(1) Background: The realization of appropriate aortic replicas for in vitro experiments requires a suitable choice of both the material and geometry. The matching between the grade of details of the geometry and the mechanical response of the materials is an open issue that deserves attention. (2) Methods: To explore this issue, we performed a series of Fluid–Structure Interaction simulations, which compared the dynamics of three aortic models. Specifically, we reproduced a patient-specific geometry with a wall of biological tissue or silicone, and a parametric geometry based on in vivo data made in silicone. The biological tissue and the silicone were modeled with a fiber-oriented anisotropic and isotropic hyperelastic model, respectively. (3) Results: Clearly, both the aorta’s geometry and its constitutive material contribute to the determination of the aortic arch deformation; specifically, the parametric aorta exhibits a strain field similar to the patient-specific model with biological tissue. On the contrary, the local geometry affects the flow velocity distribution quite a lot, although it plays a minor role in the helicity along the arch. (4) Conclusions: The use of a patient-specific prototype in silicone does not a priori ensure a satisfactory reproducibility of the real aorta dynamics. Furthermore, the present simulations suggest that the realization of a simplified replica with the same compliance of the real aorta is able to mimic the overall behavior of the vessel.

## 1. Introduction

Studies on cardiovascular diseases (CVDs) as well as the assessment of cardiovascular devices are often based on in vitro tests designed to reproduce the real environment of interest as realistically as possible. Many examples are reported in the literature, e.g., for the design and assessment of prostheses, surgical techniques [1], and, in general, the evaluation of hemodynamics in the ventricle and through the valves [2,3,4], and along the aorta and other large vessels [5]. Some studies, in particular, focus on the examination of both artificial and native vessels, to explore their similarities and/or discrepancies [6].

To represent the physio-pathology suitably, these models should describe with sufficient details (i) the anatomy of the problem, (ii) the mechanical response of both organs and tissues and blood, and (iii) the boundary conditions characterizing the phenomenon.

Over the last decade, the above aspects have benefited from the improvement of high-resolution imaging analysis and additive manufacturing [7,8]. Nowadays, high-resolution imaging acquisition, which can be easily post-processed to provide CAD models of the solid parts, is used to recreate patient-specific (PS) anatomy [9]. Moreover, the new frontiers of 3D printing facilitate the realization of realistic replicas of the anatomical districts, reducing both the time and cost of the manufacturing process, and then their use in in vitro experiments [10,11].

Although a realistic representation of the geometry can be easily obtained, the modeling of biomechanics and fluid dynamics is still a challenge issue [12,13]. Often, in fact, patient-specific phantoms, which are developed for studying flow patterns in the presence of pathologies, such as aneurysms, are made of materials that allow optical access inside the prototype. Usually, the materials are glass, Plexiglas, and silicone, i.e., materials that partially reproduce the characteristics of the biological tissue. Thus, the strong differences between the anatomy and the material properties of the prototypes raise some concerns on the opportunity to make patient-specific replicas, favoring the use of facilities with ex vivo organs [14,15].

In this framework, the present study aims at answering the following questions: can a silicone prototype suitably mimic a biological aorta? To what extent do the anatomical characteristics have to be detailed?

To give an answer to the raised questions, through Fluid–Structure Interaction (FSI) simulations, we compare three models of the aorta, obtained by combining two different geometries and two different wall materials. The aim is to assess the reproducibility of the physiological aorta (made of biological tissue) in silicone replicas. The choice of numerical analysis lies in the following advantages associated with the present FSI approach. First, we can estimate a large number of output variables on the whole domain and, accordingly, extend the comparison to quantities that cannot be inferred through in vitro experiments. Second, we avoid any problems related to the feasibility of the making process of the phantom. Third, we can prescribe the ideal working conditions favoring the comparison of the results.

The results will assist the choice of the most proper aortic arch anatomy to be adopted for a silicone phantom to be housed in a pulse duplicator for the in vitro analysis of aortic hemodynamics.

## 2. Materials and Methods

### 2.1. Description of the Numerical Models

**Figure 1:**
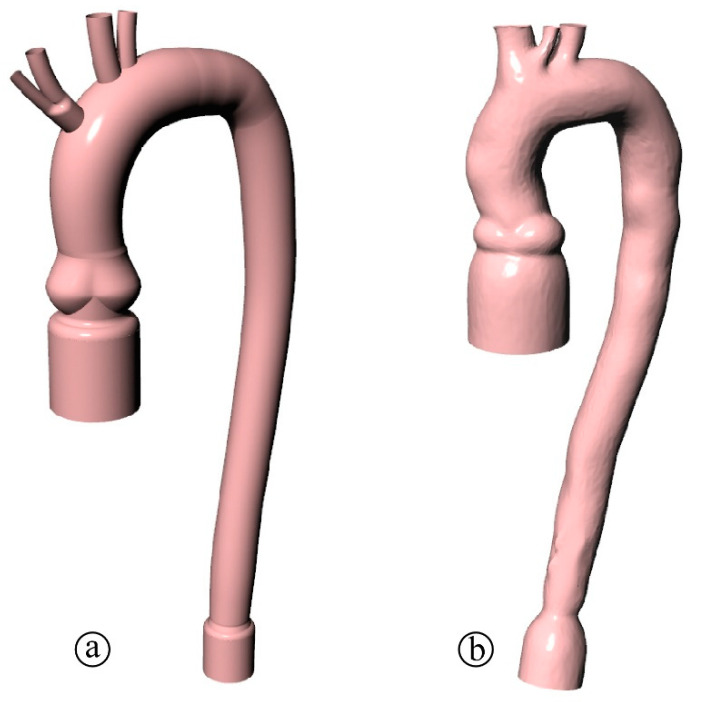
Anatomy of the phantoms: (**a**) parametrized and (**b**) patient specific aortic arch.

To study the effect of the geometry of a silicone phantom, we collected the aortic arch geometry of 20 patients that underwent an MRI test at the Padova University Hospital. Informed consent was obtained from all subjects involved in the study. From the clinical data, first, we exacted a parametrized (PM) geometry (Figure 1a) obtained as the averaged and smoothed geometry of the 20 patients with the double curvature being simplified into a planar arch. Second, we selected a patient-specific (PS) geometry of a 56-year-old woman (Figure 1b) in which the 3D curve of the arch is maintained. Both domains (PM and PS) display the peculiar elements of the real aorta, such as the Valsalva sinuses, the aortic branches, and the not constant vessel size. Since the two aortas are prototypes for in vitro experiments, tapered connections are included at the extremities to facilitate the housing into a pulse duplicator. Moreover, we remove the branches and do not model the aortic valve to ease the numerical simulations and facilitate the comparison of different aortic dynamics.

Two constitutive models are adopted for vessel walls to perform FSI simulations and compare the effects of different mechanics on the hemodynamics.

The isotropic hyperelastic model proposed by Ogden et al. [16] (hereinafter the Ogden model) is the simplest constitutive model adopted in this study. The potential strain energy density W is:

(1) $$W=\frac{2\mu }{\alpha^{2}}\left(\lambda_{1}{}^{\alpha }+\lambda_{2}{}^{\alpha }+\lambda_{3}{}^{\alpha }-3\right)+\frac{1}{D}{\left(J-1\right)}^{2},$$

where $\lambda_{1}$, $\lambda_{2}$, and $\lambda_{3}$ are the deviatoric principal stretches; μ and α are two constitutive parameters, D is a constitutive parameter related to the material compressibility, and J is the volumetric ratio. This model is already implemented in the library of the commercial software Abaqus Standard implicitly adopted here, and it is generally used to model the behavior of rubber or silicone, i.e., two materials typically adopted to make aortic phantoms.

The second constitutive scheme is that proposed by Holzapfel et al. [17] (hereinafter the Holzapfel model). According to this model, the material is hyperelastic and anisotropic, as a result of elastic fibers inserted in an isotropic matrix and oriented according to preferential directions. The form of the strain energy density for this composite material writes:

(2) $$W=C\left(I_{1}-3\right)+\frac{1}{D}\left(\frac{J^{2}-1}{2}-\ln J\right)+\frac{k_{1}}{2k_{2}}\overset{N}{\underset{\alpha =1}{\sum }}\left\{\exp[k_{2}\langle E_{\alpha }\rangle {}^{2}]-1\right\}$$

with

(3) $${\bar{E}}_{\alpha }=k\left(I_{1}-3\right)+\left(1-3k\right)\left(I_{4\left(\alpha \alpha \right)}-1\right).$$

where C is a material parameter related to the matrix shear stiffness, $k_{1}$ and $k_{2}$ express the fiber stiffness and the uncrimping phenomena, and N is the number of fiber bundles. The model assumes that the directions of the fibers within each bundle are dispersed around a mean preferred direction. The parameter κ describes the level of dispersion in the fiber directions and the quantity $E_{\alpha }$ characterizes the deformation of the bundle of fibers in the mean direction α. This model is suitable to simulate the real arterial wall [18,19], which is divided into three layers, i.e., the tunica intima, the tunica media, and the tunica adventitia, which lend the anisotropic behavior to the vessel. As we describe with more detail in Section 2.2, we calibrate the Holzapfel model with data collected by tests carried out on a porcine aorta, here assumed as a surrogate of the human aorta.

Finally, the blood is assumed Newtonian and incompressible, i.e., flow inside the vessel is modeled according the Navier Stokes equation:

(4) $$\nabla p=\rho \left(g-\frac{dv}{dt}\right)+\mu \nabla^{2}v.$$

The left-hand side term of (4) is the force due to the pressure, p; the right hand-side terms are the gravity force, g, the inertial force, and the viscous force, where v is the fluid velocity, ρ the fluid density, and μ the dynamic viscosity. In the fluid simulations, performed with the Abaqus CFD module, blood density and dynamic viscosity are assumed equal to ρ = 1025 g/cm^3^ μ = 3.5 × 10^−3^ Pa·s, respectively.

**Figure 2:**
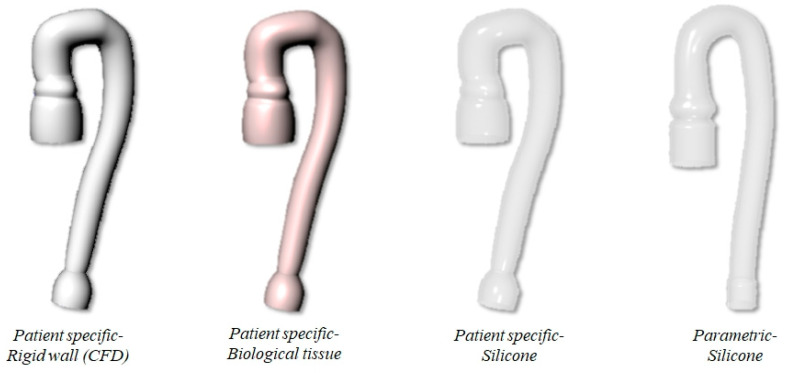
Sketch of the four aortic arch models: patient-specific anatomy with rigid wall, biological tissue and silicone, and parametric aorta in silicone.

The two aortic geometries and the two material models described above are combined to investigate three different FSI scenarios. In the first scenario, we consider the PS aorta and Holzapfel anisotropic constitutive model to simulate the biological tissue (PSB model). In the second scenario, PS geometry is made of silicone and the isotropic constitutive model is used (PSS model). In the last scenario, it is considered that the parametrized aorta PM is made of silicone (PMS model). It is important to underline that the PSB case resembles both the anatomy and the material of the real aorta, whereas the second and third case can be seen as in silico models of two possible in vitro phantoms of the problem. Finally, an additional simulation is carried out by solving the PS geometry under solid wall conditions (i.e., CFD case). The four study cases are summarized in the scheme of Figure 2.

For the comparative purpose of the present analysis, after a short mesh sensitivity study not shown here, we adopted the mesh density giving a good balance between accuracy and computational cost. In the PS model, the solid and fluid domains are represented indicatively by 123,000 tetrahedral linear elements and 124,000 hexahedral linear elements, while the PM domains are discretized by 159,000 tetrahedral linear elements and 118,000 hexahedral linear elements. The thickness of the vessel is modeled through a structured grid of 8 elements to describe with sufficient accuracy the stress condition in the wall. A structured 3D grid is generated for the CFD domain to enhance the convergence of the solution and thus reduce the computational costs. The two models are coupled using the co-simulation function of Abaqus, with the solid and fluid domains interacting through the contact surface, i.e., the internal lumen of the aorta. The FSI scheme implemented is two-way: the fluid exerts pressure on the wall deforming the aorta, and, at the same time, the compliant structure of the vessel affects the fluid flow by varying the lumen cross-section.

In all simulations, we consider the same boundary conditions, summarized as follows:

- Dirichlet conditions in portions of the solid domain, i.e., null displacements of the nodes;
- Neumann conditions at the inlet of the fluid domain, i.e., flow condition at the aortic inlet;
- Dirichlet conditions at the outlet of the fluid domain, i.e., pressure condition at the phantom outlet.

**Figure 3:**
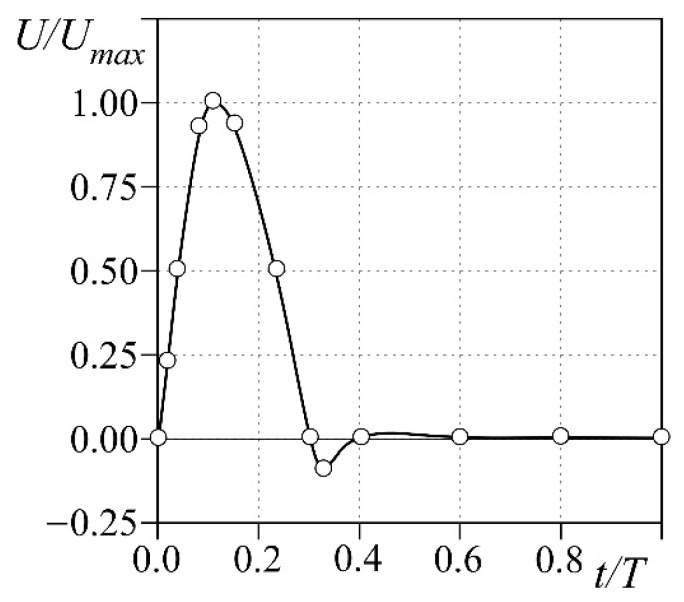
Flow condition prescribed at the inlet of the CFD domain.

Specifically, in the structural domain, we prevent any displacement of the nodes at the tapered connections consistently to the constraints on the real phantom when it is lodged into the pulse duplicator. The external pressure is set to zero so that the only forces soliciting the structural domain are due to the fluid flowing into the vessel. For the CFD simulation, we prescribe the flow ejected by the ventricle through the aortic valve as the inlet boundary condition (Figure 3). The flow is pulsatile with period *T* = 1 s (i.e., the heart rate is *HR* = 60 bpm), and a systolic fraction of 37% of the cycle; a backward flow whose maximum equals 10% of the peak is also present at end systole. The maximum flow *U_max_* is chosen so that the stroke volume ejected in each cycle is *SV* = 60 mL. At the outlet of the descending aorta, a constant pressure of 90 mmHg is set, whereas the fluid is initially at rest. We also prevent any displacement along the flux direction of the inlet and outlet surface. The overall duration of the simulation is 5.0 s, to achieve regular periodic flow into the vessel.

### 2.2. Materials Characterization

Preliminary analyses are carried out to evaluate the parameters of the structural models described in Section 2.1. Specifically, the Ogden model is calibrated to mimic PROCHIMA Cristal Rubber silicone, which is commonly adopted in blood vessel prototyping. Biaxial tests on five 3 × 3 cm^2^ samples are performed, and the averaged values of measured stress and strain are used to compute the Ogden coefficients through the algorithm provided by Abaqus.

With respect to the Holzapfel model calibration, a more complex procedure is required. First, uniaxial tensile stress tests are carried out on 4 samples of a porcine aorta obtained from a local butcher by means of a BOSE ElectroForce tensile-testing machine (no ethical statement was required). Dog bone specimens with a central rectangular region of 5 mm length, 3 mm width, and thickness in the range of 2–2.5 mm are solicited with a velocity of deformation equal to 0.5 mm/s. The deformation is determined as the ratio between the displacement of the clamps and the initial length of the specimen. The test is carried out up to deformations of 100%, i.e., a value larger than the maximum expected in our simulations. Dog bone specimens are cut in both the longitudinal and circumferential direction of the sampled aortas to account for the real tissue anisotropy and tested. The parameters of the Holzapfel model are then computed by performing 3D numerical tests specifically built up with Abaqus to reproduce the BOSE experiments. Both longitudinal and circumferential dog bone virtual samples are reproduced and schematized as a single layer with two families of collagen fibers oriented at ±ϑ with respect to the tangential axis of the vessel. To reconstruct the strain–stress curve of the uniaxial in vivo experiments, we prescribe the same displacements induced by the tensile-testing machine to the clamped regions of the virtual specimens. A trial-and-error procedure is run for the virtual sample until the numerical results fit to the average ex vivo measures.

**Figure 4:**
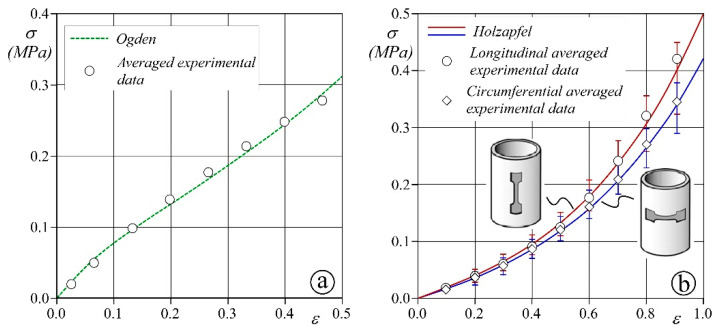
Constitutive model characterization of the Ogden (dashed green line, panel (**a**)) and Holzapfel (red and blue solid line for the longitudinal and circumferential direction, respectively, panel (**b**)) models. (**a**) The white circles show the average experimental data of biaxial stress tests of 5 samples of PROCHIMA Cristal Rubber silicone. (**b**) The white circles and white diamonds show the averaged experimental data of 4 tensile stress tests of porcine tissues along the circumferential and longitudinal directions, respectively. Red and blue bars show the maximum and minimum stress measured in the experiments to the longitudinal and circumferential direction, respectively.

The results of the mechanical model calibration process are reported in Figure 4 and show that the measured stress–strain curves compare favorably with the behavior obtained from the theoretical schemes. The estimated coefficients of the two models are summarized in Table 1.

Further numerical tests are run to determine the wall thickness, *s*, to be adopted for the phantoms, either of silicone or porcine tissue, to properly simulate the compliance of the real vessel. A pipe of length equal to 50 mm, diameter equal to 30 mm, and constant wall thickness in the range 1–4 mm is filled with stationary fluid at pressure $p_{0}$ = 80 mmHg. Starting from the above unstressed conditions (pipe inner volume: V_0_ = 35.340 × 10^3^ mm^3^), the fluid pressure is increased from $p_{0}$ up to 120 mmHg, i.e., within the physiological aortic pressure range. The pipe compliance, C, is then calculated as *C* = ΔV/Δp, ΔV being the difference between the final and the initial inner volume. Finally, the wall distensibility $AD=C/V_{0}$ is computed.

**Figure 5:**
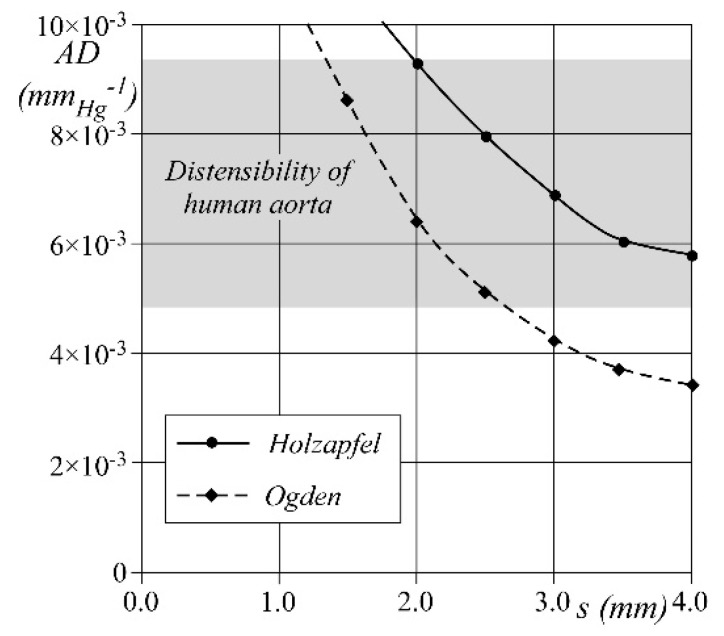
Comparison of numerical aortic distensibility between the two constitutive models; black diamonds and black circle are computed AD respectively, for the Ogden (silicone) and Holzapfel (porcine tissue). Grey area shows the range of in vivo human AD measured in young healthy patients [20].

The results obtained for both silicone and porcine tissue pipes are reported in Figure 5 as a function of the tested wall thickness s. When s holds constant, the silicone wall shows a lower distensibility than the porcine one. However, when the wall thickness is between 2 and 2.6 mm, both materials show a distensibility within the physiological range around 5 ÷ 9.1/mmHg. Accordingly, we set the value s = 2.5 mm for both porcine and silicone phantoms, regardless of the constitutive material.

## 3. Results and Discussion

**Figure 6:**
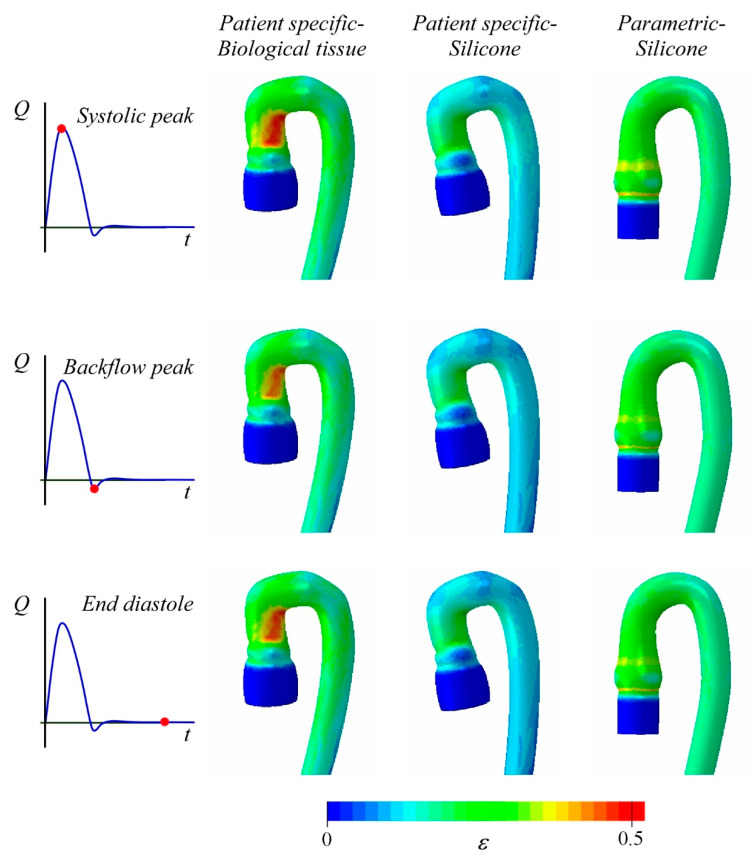
Maximum principal strain computed for the PSB model (column on the **left**), the PSS model (column at **center**), and PMS model (column on the **right**) at three time instants: the systolic peak (first row), the backflow peak (second row), and at the end diastole (third row).

Figure 6 shows the strain field computed for the arch of the three deformable aortas in three peculiar time instants of the cardiac cycle, namely, the systolic peak, the maximum backflow condition at the end systole, and the rest condition at the end of diastole. The observed pattern mainly depends on the anatomy of the arch. Both the PS geometries show the maximum deformation in the same region of the intrados at the end of the ascending aorta [21], whereas the PM geometry has nearly uniform deformation along the arch. In the latter case, the maximum is achieved on the basis of the Valsalva sinuses in the form of an annular ring.

The Holzapfel model describes an appreciably softer constitutive behavior than the Ogden model (see Figure 4). The resulting effects are clearly visible in the contour plot of Figure 6. The two PS anatomies have the same geometrical configuration and show a similar strain pattern; however, the values of the principal strain, ε, differ significantly between the two models due to the higher distensibility of the porcine tissue (Holzapfel law) than the silicone (Ogden law). In the PSB, the maximum strain value is within the range ε_max_ = 40–45%, whereas in the PSS aorta ε_max_ = 30%. Moreover, it is interesting to note that the parametric silicone aorta (PMS) shows an average strain closer to the PSB aorta (porcine tissue) than the PSS case (silicone). This may be likely due to the most complex geometry of the arch of the PS case, which hinders the vessel deformation.

**Figure 7:**
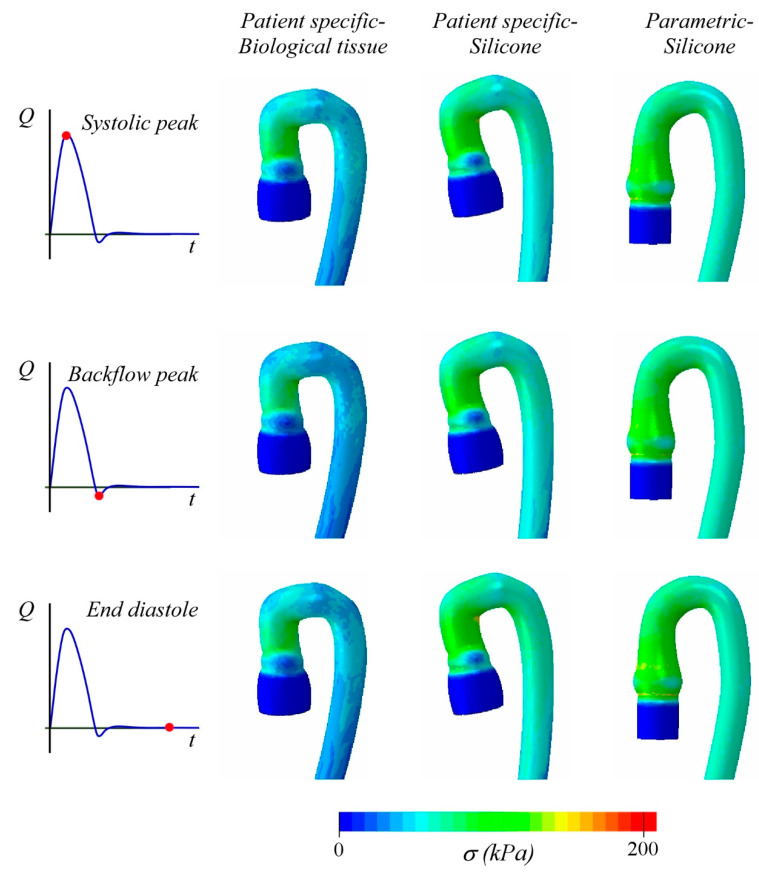
Principal stress corresponding to the strain field reported in Figure 6.

Figure 7 shows the Von Mises stress σ distribution, corresponding to the strain condition of Figure 6. The porcine tissue phantom displays wider areas with low values of σ than the other two cases, due to the material behavior that is softer than the silicone one used for PSS and PMS models. For instance, considering the two PS geometries, the maximum Von Mises stress is computed at arch intrados, and it is equal to 160 and 200 kPa for the porcine and silicone aorta, respectively. It is important to note that, in the parametric case, although the average stress is comparable with that of PSS, the maximum stress estimated at the intrados is σ = 120 kPa. We can ascribe this relatively low value of stress to the simpler arch geometry.

**Figure 8:**
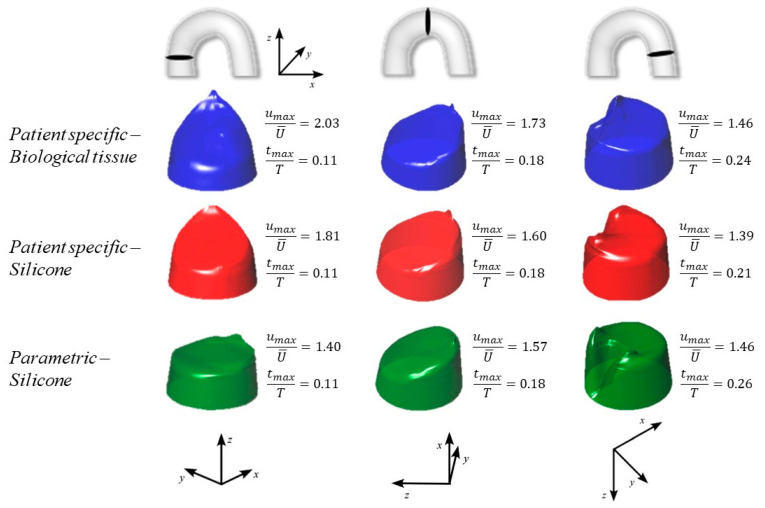
Cross-sectional velocity distribution of the systolic peak in the inlet (first column), in the middle (second column), and in the outlet (third column) of the aortic arch. The blue, red, and green color indicates the PSB case, the PSS case, and the PMS case, respectively.

The nature of the curvature of the arch, i.e., planar (PM) or 3D (PS), significantly affects the flow, as well. In Figure 8, we summarize the axial velocity distribution at peak systole at the inlet, the middle, and the outlet sections of the arch. The velocity profile is normalized by the average local velocity, $\bar{U}$, in order to minimize the effect of the lumen size and effectively compare the results. In the PM geometry, the flow at the inlet is rather uniform across the section: the maximum velocity, $u_{max}$, which establishes near the intrados, results to be just 40% higher than the average velocity. On the contrary, in the PS geometry, the velocity near the intrados is observed to be almost twice the average flow. The above difference in the velocity distribution between the two geometries diminishes as we move downstream, where analogous velocity profiles are estimated for the three cases (see the middle and the outlet section in Figure 8). The CFD simulations agree with the expected results, as remarked by the wake clearly visible in the outlet intrados [22,23].

Some interesting comments concerning the time of peak velocity, $t_{peak}$, also arise. The same $t_{peak}$ is estimated at the inlet and at the middle section (0.11 s and 0.18 s, respectively) in all three scenarios. Conversely, at the outlet section, the peak flow is recorded at different times. This is mainly due to the compliance of the arch, i.e., to a combination of the constitutive model and the geometry. If we compare the two PS anatomies, the peak is anticipated in the silicone aorta (t = 0.21 s), in which the volume variation, and hence C, is relatively small compared to the porcine case, where the peak time occurs at t = 0.24 s. The maximum delay is computed for the parametric geometry (t = 0.26 s), but this result is with high probability due to the longer arch of the PM model.

**Figure 9:**
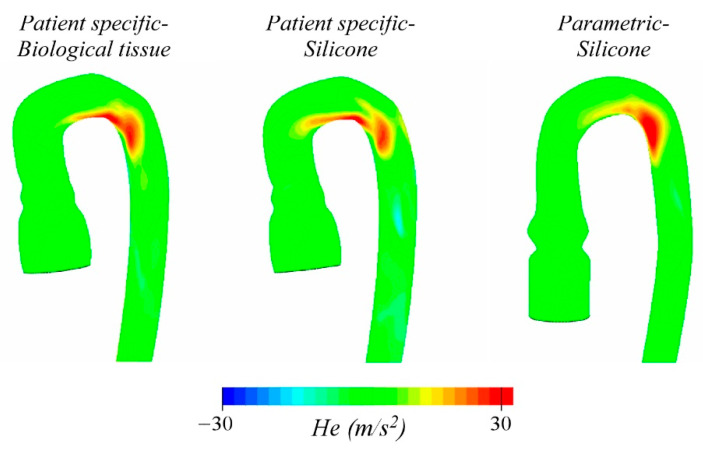
Helicity field computed for the three models at the time *t*/*T* = 0.18.

Figure 9 shows the helicity, He, i.e., the scalar product between the velocity and the vorticity vectors, calculated at t/T = 0.18. This parameter, strictly related to blood pulsatility, is useful to characterize the helical flow through the arch and seems to be a predictor of diseases such as aortic aneurism and dissection. The intensity of He is maximum at the arch [24] and varies in the three cases between 20 and 30 m/s^2^. Some differences in the spatial distribution can be observed, even if to a more minor extent than before. Presumably, they are due to the anatomy of the arch: both PSB and PSS show high helicity along the intrados of the arch up to the beginning of the descending aorta, while in the PMS model, it is this latter region the one that results to be mainly interested.

It is also worth examining the behavior of global features of the aortic flow as obtained in the three tested models. This kind of information, in fact, may be useful to characterize the overall response of phantoms adopted for the in vitro assessment of global hemodynamics of cardiovascular devices, e.g., stents, occluders, and valves [1,25].

**Figure 10:**
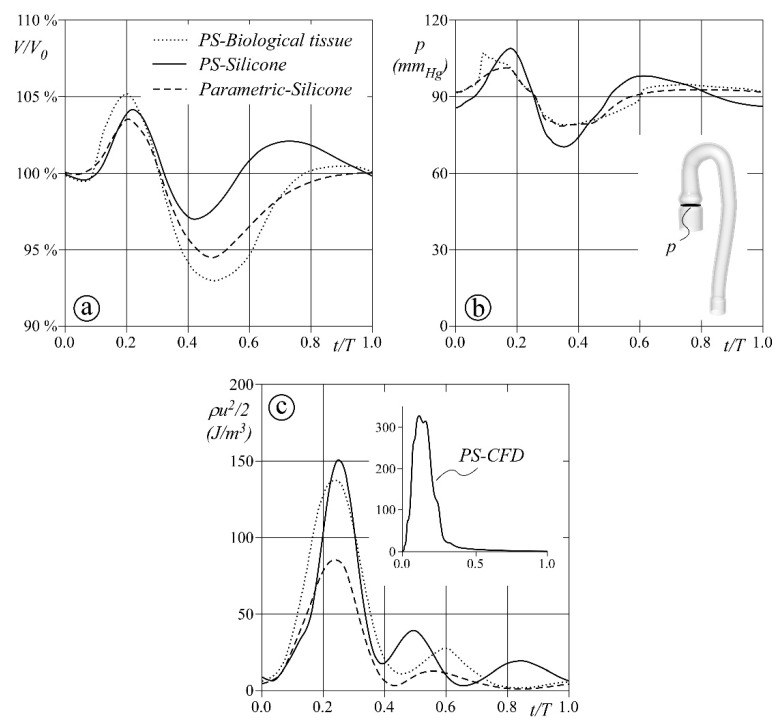
Global performance of the three aortas during the cardiac cycle. (**a**) Total volume variation; (**b**) mean pressure at the aortic valve inlet; and (**c**) mean flow kinetic energy.

Figure 10 reports the behavior in one cardiac cycle of the inner volume $V/V_{0}$, the pressure p at the aortic annulus, and the mean kinetic energy in the whole domain $\rho u^{2}/2$. Panel a, in particular, shows how the aortic models accumulate and release blood: during the systolic ejection, the PSB phantom increases its volume by about 5%, not so far from the relative increments of 4% and 3.5% estimated for the PSS and PMS phantoms. On the contrary, the phantoms’ dynamics significantly change during the diastole. A contraction is clearly visible in the early stage and mainly affects the PSB anatomy and the PMS aorta. However, in the next stage, they both gradually recover their initial volume $V_{0}$, whereas PSS results to be less effective in damping volume oscillations. The estimated pressure at the aortic annulus (Panel b) reflects the reduced compliant capacity of PSS phantom. The pressure wave shows oscillations consistent with the volume variations, and large up to ±20 mmHg (maximum and minimum pressure close to 110 and 70 mmHg, respectively). In the other two modeled scenarios, the pressure variation is lower and ranges between 80 and 100 mmHg. Finally, Panel c shows the mean kinetic energy through the entire domain, which can be seen as a measure of blood dynamic effects on the vessel structure as well as on devices possibly deployed along the vessel itself. This parameter results to be quite significantly affected by the model geometry, in particular during the systolic ejection. At the time of peak flow, $\rho u^{2}/2$ is found to be about 150 J for both PSB and PSS, while it halves for the PMS model. Moreover, during the diastolic phase, oscillations are found for all three models, which seem more sensitive to the wall material and are consistent with the behavior of the volume, as expected. Notice also, that when solving the CFD model for the PS anatomy (see the inset in Panel c), the kinetic energy varies during the systolic ejection only and reaches a maximum value more than double the FSI result. Wall deformability also significantly affects the phase delay of both pressure and flow pulses propagation, irrespective of the tested model. For instance, the maximum mean kinetic energy is simultaneous to the inlet peak flow in the CFD simulation (t = 0.18 s) and ranges from 0.23 to 0.25 s in the three deformable aortas.

### Study limitations and Future Developments

Some study limitations can be highlighted. First, in the present study, we ease the Dirichlet condition prescribing a constant pressure at the outlet, i.e., a boundary condition far from the time-dependent physiological pressure in the descending aorta. Although such a simplification still allows one to consistently compare the different aortic models as here presented, a more suitable representation of the outlet boundary condition would enhance the computed hemodynamics. To this aim, the 3D models of the aorta could be coupled to a lumped parameters model of the downstream vascular circulation (see, e.g., [26,27,28]). Upper body circulation could also be accounted for by including one further 0D model at the level of the aortic arch, i.e., at the carotid junction location. From this perspective, it is worth recalling that 0D models of blood circulation have been proven not only to be robust and less time-consuming than 3D ones, but also suitable to simulate flows and pressures in diseased conditions [29,30,31].

Second, the material characterization, especially the definition of the biological tissue properties, is determined on a limited number of samples. Although a consistent statistic due to a large number of tested specimens can enhance the definition of the strain–stress curves of the aortic wall, in ex vivo testing, the properties of the aortic tissue are, however, altered and may significantly differ from the in vivo conditions. To overcome this drawback, a possible solution consists of assessing the biomechanical properties through non-invasive in vivo measures of aortic wall displacements [32].

The introduction of the aortic valve is another possible step to refine the present models. However, the modeling of moving and deformable leaflets is highly demanding. For this reason, the implementation of the aortic valve should be carefully considered, mainly in relation to the scopes of the research. In fact, if it is true that aortic valve dynamics significantly affect the flow in the Valsalva sinuses [33], it is also recognized as a secondary factor on the hemodynamics of the arch and the descending aorta, at least in the absence of valvular diseases.

In general, future developments of both in silico and in vitro models of the aorta should carefully consider the grades of details that one wants to simulate according to the study purposes and users [34].

## 4. Conclusions

In the present study, we numerically compare three different models of the aortic vessel, built up by combining two distinct anatomies and two different deformable materials. The first model (PSB) may seem to resemble the real aorta more closely than the other two, since it is based on an MRI patient-specific geometry and an anisotropic model calibrated with ex vivo data from porcine aortic tissue. In the other two models (PSS and PMS), the considered material is isotropic and hyperelastic, and reproduces the mechanical properties of the silicone usually adopted for in vitro phantoms. The two silicone models differ in their geometry, since one model uses MRI patient-specific data while the other has a parametric geometry based on averaged in vivo measures.

The results indicate that anatomy is the main factor that influences the deformation pattern of the aortic vessel, and the material significantly affects the magnitude of the strain field. Flow velocity distribution is quite sensitive to local geometry, which, on the contrary, seems to play a minor role on the helicity along the arch.

However, the overall behavior seems to be mainly governed by the model compliance, which results from the combination of the geometry, the constitutive model, and the applied constraints. In the present analysis, both the volume and pressure variations computed for the PMS case in one cardiac cycle satisfactorily agree with those estimated for the PSB case.

The above observations have potential implications on the criteria to be followed when prototyping aortic vessels for in vitro facilities. In applications based on the quantification of global parameters, the use of a patient-specific approach may be unnecessary if it is limited to the vessel anatomy and, on the other hand, current research is still working on materials that closely resemble the biological tissue, either treated as population- or patient-specific. On the contrary, the adoption of both simplified geometry and rubber-like material may satisfactorily mimic the real vessel, as our results seem to suggest, at least when the investigation is focused on population-specific questions rather than on seeking answers and solutions tailored for that given patient.

## Figures and Tables

**Table 1:** Estimates of the parameters for the silicone (Ogden model, Equation (1)) and the biological tissue (Holzapfel model, Equations (2) and (3)).

**Ogden**					
*μ*_1_		*α*_1_		*D*_1_ (MPa^−1^)	
1.73 × 10^−1^		4.39		1.193	
**Holzapfel**					
*C* (MPa)	*k*_1_ (MPa)	*k*_2_	*k*	*D* (MPa^−1^)	*θ*°
2.89 × 10^−2^	1.20 × 10^−1^	0.4	0.25	0.7	27

## Data Availability

The data presented in this study are openly available at http://researchdata.cab.unipd.it/id/eprint/488 (accessed on 11 May 2021).
